# Edge effects and social behavior in three platyrrhines

**DOI:** 10.1002/ajp.23610

**Published:** 2024-02-24

**Authors:** Laura M. Bolt, Jenna L. Owens, Madison Taylor Grant, Elizabeth M. C. Coggeshall, Dorian G. Russell, Carrie Merrigan‐Johnson, Zachary S. Jacobson, Zachary T. Schmidt, Francesca V. E. Kaser, Amy L. Schreier

**Affiliations:** ^1^ Department of Anthropology University of Toronto Mississauga Mississauga Canada; ^2^ Maderas Rainforest Conservancy Miami Florida USA; ^3^ Department of Anthropology University of Texas at San Antonio San Antonio Texas USA; ^4^ Department of Anthropology Graduate Center of the City University of New York New York New York USA; ^5^ Department of Anthropology Indiana University Bloomington Bloomington Indiana USA; ^6^ Department of Environmental Science American University Washington District of Columbia USA; ^7^ Department of Anthropology and Archaeology University of Calgary Calgary Canada; ^8^ Department of Psychology University of Wisconsin Madison Wisconsin USA; ^9^ Department of Biology Regis University Denver Colorado USA

**Keywords:** *Alouatta palliata*, anthropogenic edge effects, *Ateles geoffroyi*, *Cebus imitator*, Costa Rica

## Abstract

Social behavior is a key adaptation for group‐living primates. It is important to assess changes to social behavior in human‐impacted landscape zones to better understand the impact of anthropogenic disturbance on primate species. We investigated social behavior rate and type in three species of platyrrhines across 100 m anthropogenic edge and interior zones of a fragmented forest in Costa Rica, La Suerte Biological Research Station (LSBRS). Following results from other sites, we predicted that spider monkeys (*Ateles geoffroyi*), capuchin monkeys (*Cebus imitator*) and howler monkeys (*Alouatta palliata*) would show lower rates and fewer types of social behavior in forest edge compared to interior. We collected 1341 h of instantaneous focal data from 2017 to 2023 across the three monkey species. We found mixed support for our predictions, with spider and capuchin monkeys modifying some but not all aspects of social behavior across forest zones at LSBRS. Spider monkeys had lower rates of social behavior and capuchin monkeys performed different types of social behaviors in forest edge compared to interior at LSBRS. In contrast, howler monkeys did not modify social behavior. Two out of three platyrrhine species altered their social behavior when in anthropogenic edges, indicating behavioral adjustment when in human‐altered habitat areas at LSBRS.

## Research Highlights

- Central American spider monkeys (*Ateles geoffroyi*) have a lower rate of social behaviors in anthropogenic forest edge compared to forest interior.
- White‐faced capuchin monkeys (*Cebus imitator*) perform different social behaviors in forest edge compared to interior.
- Mantled howler monkeys (*Alouatta palliata*) do not modify social behavior across forest zones.

## Graphical abstract

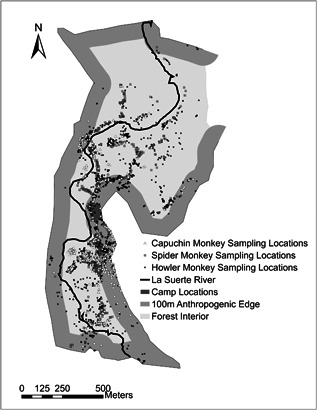

**Abbreviation**
LSBRS: La Suerte Biological Research Station

## 1 INTRODUCTION

In group‐living mammals such as primates, a variety of factors influence behavior, including habitat characteristics. Forest size and vegetation density, for example, impact primate ranging and feeding, and likely also impact social behavior (Arroyo‐Rodríguez & Dias, 2010; McKinney, 2010; Ukizintambara, 2010). When a forest is fragmented by humans, the area bordering clear‐cut regions is subject to edge effects, altering abiotic and biotic characteristics compared to forest interior (Chen et al., 1999; Laurance & Yensen, 1991). Trees are typically smaller, vegetation is less dense, and there is lower biodiversity in vegetation for forest interior‐adapted plant species (Didham & Lawton, 1999; Tabarelli et al., 2008). This in turn impacts animal species, with the reduced food and habitat available causing animals to avoid forest edges in many cases (Arroyo‐Rodríguez & Dias, 2010; Estrada et al., 1999a), and to potentially alter their behavior when in this region. Around 20% of the world's forests exist within 100 m of edges (Haddad et al., 2015) and more than half of living primates are declining in population size due to human‐caused habitat loss (Estrada et al., 2017; Mittermeier et al., 2012). With social behavior being a key adaptation for group‐living mammals (Kappeler et al., 2013) and with forest edges being such a common part of primate habitats worldwide, it is therefore salient to investigate how this human‐caused habitat alteration impacts the social behavior of primates.

A small number of previous investigations reported that when primates are in the edge areas of fragmented forests, their social behavior is altered. For example, L'Hoest's monkeys (*Cercopithecus lhoesti*) living in a group at the forest edge performed significantly less social behavior (defined as allogrooming, playing, agonism, or copulation) compared to monkeys living in forest interior (Ukizintambara, 2010). This lesser amount of social behavior when in forest edge also impacted group cohesion, with monkeys in forest edge being more widely spaced from one another compared to monkeys in forest interior (Ukizintambara, 2010). Similarly, Weddell's saddle‐back tamarins (*Leontocebus weddelli*) performed significantly less social behavior (allogrooming) in forest edge compared to forest interior (de Vries, 2017). However, this trend is not universal. The emperor tamarin (*Saguinus imperator*) preferred the forest edge region but there was no difference in social behavior (allogrooming) across edge and interior zones (de Vries, 2017), while the Coquerel's sifaka (*Propithecus coquereli*) preferred the forest interior but also showed no difference in social behavior (allogrooming, playing) across forest edge and interior zones (McGoogan, 2011). As Ukizintambara (2010) identified, further investigation of how forest edges impact the social behavior of primates is needed.

There is more research investigating the social behavior of primates living in fragmented forests or other disturbed habitats compared to their social behavior in continuous forests or pre‐disturbance. In general, primates performed a smaller proportion of social behaviors out of total activity budget when living in fragments compared to when in continuous forest (strepsirhines: diademed sifaka [*Propithecus diadema*], Irwin, 2007; platyrrhines: white‐faced capuchin monkey [*Cebus imitator*], McKinney, 2010; black howler monkey [*Alouatta pigra*], Rangel‐Negrín et al., 2016; mantled howler monkey [*Alouatta palliata*], Clarke et al., 2002; Juan et al., 2000; McKinney, 2019), and before a hurricane compared to following this habitat disturbance (black howler monkey, Behie & Pavelka, 2005; Pavelka et al., 2003). Primates also performed social behavior at lower rates (mantled howler monkey, Clarke et al., 2002; Juan et al., 2000; McKinney, 2019), and performed fewer different types of social behaviors when in fragmented forest compared to continuous forest (black howler monkey, Negrín et al., 2016; white‐faced capuchin monkey, McKinney, 2010).

However, social behavior findings in fragments were also mixed. Some monkeys showed no differences in social behavior rate or duration across fragmented and continuous forests (Central American spider monkey [*Ateles geoffroyi*]: allogrooming, playing, agonism, Chaves et al., 2011; red howler monkey [*Alouatta macconnelli*]: vocalizing, McKinney et al., 2015; mantled howler monkey: playing, agonism, McKinney, 2010, 2019). Other monkeys that differed in total amount of social behavior out of overall activity budget did not change the types or allocation of social behaviors performed (i.e. relative proportion of behaviors such as allogrooming, playing, agonism, vocalizing and copulation out of total social behavior activity budget) when compared across fragmented and continuous forests (mantled howler monkey: allogrooming, playing, agonism, vocalizations, copulation, McKinney, 2010, 2019; white‐faced capuchin monkey: allogrooming, playing, agonism, McKinney, 2011). Further, some species, such as the Central American spider monkey, the white‐faced capuchin monkey, and the mantled howler monkey, display conflicting results depending on the study (Central American spider monkey: Chaves et al., 2011 vs. Lindshield, 2006; white‐faced capuchin monkey: McKinney, 2010 vs. McKinney, 2011; mantled howler monkey: Clarke et al., 2002 and Juan et al., 2000 vs. McKinney, 2010, 2019), suggesting that primate responses may depend on a more complex array of factors than forest size alone, and further examination is warranted. Continued investigation of the relationship between primate social behavior and habitat alteration is needed to further understand the impact of deforestation on the behavioral ecology of primates. In particular, the effects of anthropogenic forest edge on primate social activity budgets and social behaviors are not well understood and require further study (Arroyo‐Rodríguez & Dias, 2010; de Vries, 2017; Ukizintambara, 2010).

We investigated the impact of human‐caused deforestation on social behavior rate and type in three species of platyrrhine primates living in an anthropogenically‐fragmented rainforest in Costa Rica, La Suerte Biological Research Station (LSBRS): the Central American spider monkey (*Ateles geoffroyi*), the white‐faced capuchin monkey (*Cebus imitator*), and the mantled howler monkey (*Alouatta palliata*). Previous research at other sites on all three species demonstrated that their social behavior is affected in some way by forest fragmentation. Central American spider monkeys altered their overall amount of social behavior when in disturbed compared to continuous forest (Lindshield, 2006; but see Chaves et al., 2011), while white‐faced capuchins performed a lesser proportion of social behavior out of total activity budget, and performed different types of social behaviors when in human‐altered habitat regions compared to continuous forest (McKinney, 2010). Similarly, mantled howler monkeys performed a lower proportion of social behavior (Clarke et al., 2002; Juan et al., 2000; but see McKinney, 2010). At LSBRS, previous work found differences in overall howler monkey activity budget across forest zones, with more resting and less traveling in forest edge compared to interior, although the proportion of social behavior was not assessed (Schreier et al., 2022b).

With these species‐specific examples as well as other studies generally showing that primate social behavior is altered by human disturbance or human‐disturbed landscapes (Chaves et al., 2011; de Vries, 2017; Gómez‐Espinosa et al., 2014; Negrín et al., 2016; Ukizintambara, 2010), we expected all three monkey species at LSBRS to perform less social behavior when in anthropogenic forest edge compared to forest interior regions. We predicted that all three species would show a lower overall rate of social behavior and fewer different types of social behavior when in forest edges compared to forest interior regions at LSBRS. Following previous research at other sites (McKinney, 2010; Negrín et al., 2016), we also expected to see differences in the proportions of various social behaviors (allogrooming, playing, agonism, vocalizing, copulation) performed by all monkey species across LSBRS forest zones. At LSBRS, trees are smaller and vegetation species richness and density are lower in forest edge (Bolt et al., 2018, 2019), indicating a poorer‐quality habitat for most primates (Arroyo‐Rodríguez & Mandujano, 2006; Estrada et al., 1999a; Ross & Srivastava, 1994). Following findings for the black howler monkey (Negrín et al., 2016), we predicted that when in these vegetation‐poor forest areas, Central American spider and mantled howler monkey species at LSBRS would demonstrate a smaller proportion of energetically‐costly (playing, agonism) and affiliative (allogrooming) social behaviors when in forest edge, to minimize energetic expenditure in this degraded forest zone (Bicca‐Marques, 2003; Bolt et al., 2021b; Schreier et al., 2022b). For white‐faced capuchin monkeys, who show affiliative responses to forest edges at LSBRS and are likely not negatively affected by vegetation in forest edge (Bolt et al., 2020b; Tinsley Johnson et al., 2020), we made similar predictions following past research (McKinney, 2010). We also predicted that capuchins would show different types of social behavior in edge and interior, with more affiliative (allogrooming, playing) and fewer aggressive (agonism) behaviors in forest edge, following results from white‐faced capuchin monkeys in human‐disturbed habitats (McKinney, 2010).

## 2 METHODS

### 2.1 Study site

**Figure 1:**
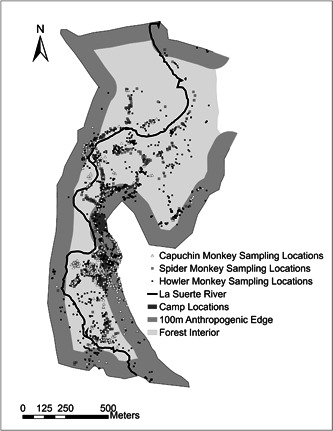
Map of La Suerte Biological Research Station showing the study area and platyrrhine behavioral sampling locations for each 30 min of sampling.

La Suerte Biological Research Station (LSBRS) is a 3 km^2^ fragment of tropical lowland rainforest located in a largely deforested region of northeastern Costa Rica, with the La Suerte river traversing northward throughout the property (Brandt & Singleton, 2018; Molina, 2015). Although LSBRS has governmental protection status and is maintained by the non‐profit organization Maderas Rainforest Conservancy (Bolt et al., 2022a; Molina, 2015), it is surrounded by cattle ranches and agricultural fields, and there are sudden landscape transitions between LSBRS's protected forest and the clear‐cut neighboring properties. Our study area consists of the 1.36 km^2^ portion of the site where research is conducted and that contains infrastructure for students and researchers (“Camp”) (Figure 1). We defined anthropogenic edge as the forest region extending 100 m from clear‐cut areas at LSBRS, which included camp as well as neighboring cattle ranches and agricultural fields (Bolt et al., 2018, 2021b; Schreier et al., 2021). LSBRS provides habitat to three monkey species: Central American spider monkeys (*Ateles geoffroyi*), white‐faced capuchin monkeys (*Cebus imitator*), and mantled howler monkeys (*Alouatta palliata*), which comprise the subjects of our study.

### 2.2 Study subjects

Central American spider monkeys are large‐bodied frugivores who live in fission‐fusion social systems (Chapman et al., 1995; Di Fiore et al., 2011). We collected Central American spider monkey social behavior data from the community of spider monkeys that ranges across both forest interior and anthropogenic edge zones at LSBRS (Bolt et al., 2022a). We could not consistently identify individual monkeys between sampling seasons, but at least 40 individual spider monkeys were present at LSBRS during each study season and we have observed and sampled them during various sampling periods (Bolt et al., 2022a; Schreier & Bolt, unpublished data). Individual spider monkeys varied in their degree of habituation, but the community has become increasingly habituated since the start of our spider monkey behavioral data collection in 2018 (Bolt & Schreier, 2023; Bolt et al., 2022a; Schreier & Bolt, unpublished data).

White‐faced capuchin monkeys are small‐bodied dietary generalist frugivores who typically live in groups of 20 or more individuals, with many groups fissioning when more than 30 individuals are present (Hogan et al., 2019; Melin et al., 2020). We collected white‐faced capuchin monkey social behavior data from three groups of monkeys comprising a population of at least 35 individuals across both forest interior and anthropogenic edge zones at LSBRS (Garber et al., 2010; Mallott, 2016; Schreier & Bolt, unpublished data). We could not consistently identify monkeys between sampling seasons due to changing group demography, but we observed and sampled two to three different groups with overlapping ranges spanning both forest edge and interior zones during each sampling period (Schreier & Bolt, unpublished data).

Mantled howler monkeys are energy‐minimizing, large‐bodied frugivore‐folivores who live in groups of 1–40 individuals (Di Fiore et al., 2011). We collected mantled howler monkey social behavior data from 11 groups that ranged across both forest interior and anthropogenic edge zones at LSBRS (Bolt et al., 2022b). These same groups have also been the subjects for other behavioral investigations at LSBRS (Bolt et al., 2020a, 2021a, 2023b; Schreier et al., 2022a), and comprise an unusually dense population of at least 149 individual monkeys or 109.5 individuals/km^2^, which is one of the highest population densities recorded at any site worldwide for this species (Bolt et al., 2022b; Schreier et al., 2023). While we could not consistently identify individual monkeys between sampling seasons due to changing group demography, we consistently observed 11 different groups across sampling seasons (Bolt et al., 2020a, 2023b; Schreier et al., 2021). Howler monkey groups were habituated and did not react to observer presence (Bolt & Schreier, 2023).

For all three monkey species, we completed focal samples on individual monkeys and rotated focal subjects between adult males, adult females, and juveniles. Infants, who rarely travel and feed independently, were not sampled. Individuals from different groups were sampled during each data collection day. We did not sample the same focal animal more than twice in 1 day, and any occasional re‐samples of the same monkey on the same day were collected at least 2 h apart (Bolt et al., 2021a, 2021b; Schreier et al., 2021). Although individual identities of monkeys were unknown in this population and some individuals were likely sampled multiple times over the study period, both within and between study seasons, we avoided re‐sampling individuals on a daily basis by keeping track of individual characteristics such as differences in group membership, ranging area and location sampled, body size, tail length and patterning, facial features, and more distinct physical characteristics when available (Schreier et al., 2021, 2022b; Schreier & Bolt, unpublished data).

### 2.3 Monkey social behavior data collection

We collected monkey social behavior data over 13 months spanning both wet and dry seasons from 2017 to 2023: May–August 2017 (capuchin and howler monkeys), May–August 2018 (spider and howler monkeys), December–January 2018–2019 (howler monkeys), June–August 2019 (spider, capuchin, and howler monkeys), December‐January 2019–2020 (spider, capuchin, and howler monkeys), December‐January 2021–2022 (spider and capuchin monkeys), June–July 2022 (spider and capuchin monkeys), and December‐January 2022–2023 (spider and capuchin monkeys) (Table 1). While more data were collected during wet seasons (May–August) compared to dry seasons (December‐January) for all three species, preliminary analyses demonstrated that trends for social behavior were consistent across seasons, so data were grouped together during analyses. We collected data daily between 500 and 1800 h, with 1–6 researchers collecting behavioral data across species during each day of sampling. Data collectors worked independently and rotated between different forest regions at LSBRS to ensure sampling of different monkey groups and representation of all forest regions. Data collectors used transect lines spaced approximately 150 m apart (Bolt et al., 2020b; Russell, 2018) as reference points for locating groups and individuals, but additionally sampled monkeys from all three species opportunistically when encountered. To assess interobserver reliability, researchers simultaneously scored monkey behaviors during pilot data collection. We achieved a 94% reliability rate before the onset of data collection within each study season (Bolt et al., 2023b; Schreier et al., 2022b).

**Table 1:** Behavioral sampling effort for three platyrrhine species at LSBRS.

Sampling season	Central American spider monkey (*Ateles geoffroyi*) behavioral sampling hours	White‐faced capuchin monkey (*Cebus imitator*) behavioral sampling hours	Mantled howler monkey (*Alouatta palliata*) behavioral sampling hours
May–August 2017	0	24	278
May–August 2018	33.5	0	458
December 2018–January 2019	0	0	48
June–August 2019	47.5	141	16
December 2019–January 2020	16.5	12	66
December 2021–January 2022	48	14	0
June–July 2022	47.5	35.5	0
December 2022–January 2023	43	12.5	0
Total data	236	239	866

We collected data using the point sampling method, using 30‐min focal animal sampling with instantaneous recordings on social and other behaviors (feeding, traveling, resting, other) taken at 2‐min intervals (Altmann, 1974; Schreier et al., 2021). Social behaviors were classified as allogrooming (run fingers/mouth along fur of another individual), playing (chasing, wrestling, exchanging objects, and other forms of interactive play), agonism (including both aggressive and submissive behaviors, such as fight, bite, lunge, attack, supplant, present rear end, and flee), vocalizing (producing audible sounds using the voice), or copulation (mating behavior consisting of intromission and/or thrusting involving any two conspecifics of any age/sex class; Schreier et al., 2022b; Teichroeb et al., 2021). Any species‐specific social behaviors were grouped into the most appropriate broader category for analysis (e.g., capuchin interindividual plant oil “fur rubbing” classified as allogrooming; McKinney, 2010). We calculated social behavior rate as the number of 2‐min scans in a particular activity per hour.

We collected a total of 1 341 h of behavioral data: 236h of behavioral data (472 30‐min focal samples) on spider monkeys (87 h in forest edge and 149h in forest interior), 239h of behavioral data (478 30‐min focal samples) on capuchin monkeys (169h in forest edge and 70 h in forest interior), and 866h of behavioral data (1732 30‐min focal samples) on howler monkeys (458 h in forest edge and 408 h in forest interior). We recorded the location of each 30‐min behavioral sample using a Garmin GPSMAP 62 s hand‐held navigator to designate whether each sample was located in 100 m anthropogenic edge or forest interior.

### 2.4 Statistical analyses

Data were not normally distributed, so we used Mann‐Whitney U tests to compare overall rate of social behavior for each species across forest edge and interior zones. We also report the mean and standard deviation, as well as visualize the data distribution for additional information, however the statistical tests are based on median values. We used two‐way Pearson chi‐squared tests (Bolt et al., 2019, 2021b) for each of the three monkey species to compare whether the social behaviors performed (allogrooming, playing, agonism, vocalizing, and copulation) showed different distributions than expected by chance across forest edge and interior zones, assuming an equal distribution of social behaviors across forest zones. As post‐hoc tests to determine which social behaviors were performed more or less than expected by chance in forest edge and interior, we examined adjusted residuals and identified z‐scores greater than ±1.97 as significantly different. We used SPSS version 29 (IBM SPSS Statistics, IBM Corporation, Armonk, N.Y., USA) for all statistical tests, and adopted an alpha level of 0.05.

## 3 RESULTS

**Figure 2:**
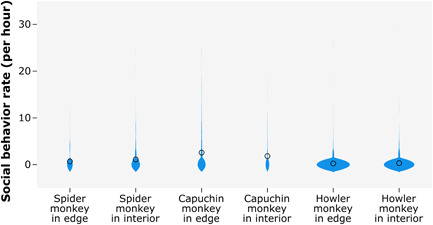
Rate of platyrrhine social behavior in forest edge versus interior at LSBRS, with violin plots showing data distributions. The width of each density curve correlates to the frequency of data points in each region, and circles indicate mean values.

Central American spider monkeys performed social behaviors during 4.2% of total behavioral sampling time, white‐faced capuchin monkeys performed social behaviors during 10.2% of sampling and mantled howler monkeys performed social behaviors during 0.98% of sampling. All species were observed performing social behaviors such as allogrooming, playing, agonism, vocalizing, and copulation across both edge and interior forest zones (Figure 1). Spider monkeys performed social behaviors at a significantly lower rate when in forest edge (mean = 0.7, SD = 1.9; median = 0, range = 0–12, *n* = 173) compared to interior (mean = 1.1, SD = 2.8; median = 0, range = 0–24, *n* = 298; Mann‐Whitney U test: U = 23613.5, z = −2.127, *p* = 0.033, *n* = 236 h, Figure 2), while capuchin and howler monkey social behavior rate did not vary across forest zones (capuchin monkeys: forest edge mean = 2.6, SD = 4.8; median = 0, range = 0–24, *n* = 338, forest interior mean = 1.8, SD = 3.6; median = 0, range = 0–18, *n* = 140; U = 22015.5, z = −1.389, *p* = 0.165, *n* = 239 h; howler monkeys: forest edge mean = 0.2, SD = 1.4; median = 0, range = 0–26, *n* = 929, forest interior mean = 0.3, SD = 1.6; median = 0, range = 0–30, *n* = 872; U = 397615.5, z = −1.491, *p* = 0.136, *n* = 866 h, Figure 2).

Despite the lack of difference in overall social behavior rate across forest zones, capuchin monkeys performed different types of social behaviors than expected based on chance when in forest edge compared to interior zones (x^2^(4) = 48.37, *n* = 498 social behaviors, *p* < 0.001, Table 2). The strength of association between variables was strong (Cramer's V test: *v* = 0.312, *p* < 0.001), and post hoc examination of adjusted residuals indicated that when adjusted for sample size, monkeys performed significantly more play behaviors and fewer agoniztic and vocalization behaviors than expected in forest edge, while performing fewer play behaviors and more agoniztic and vocalization behaviors than expected in the forest interior (z < ±1.97). Observed frequency of allogrooming and copulation behaviors did not differ from expected values across forest zones (z < ±1.97). In contrast, spider and howler monkeys performed the different types of social behaviors in the proportions expected based on chance when in edge versus interior zones (spider monkeys: x^2^(4) = 3.074, *n* = 224 social behaviors, *p* = 0.545; howler monkeys: x^2^(4) = 7.73, *n* = 206 social behaviors, *p* = 0.102).

**Table 2:** White‐faced capuchin monkey (*Cebus imitator*) types of different social behaviors performed across forest edge and interior zones at LSBRS showing percentages of different social behaviors performed and z‐scores for adjusted residual values.

	Allogrooming	Playing	Agonism	Vocalizing	Copulation
Interior forest zone social behavior (z‐score)	69.0% (−0.1)	3.2% (−4.4*)	14.3% (2.2*)	13.5% (5.3*)	0% (−1.3)
Edge forest zone social behavior (z‐score)	69.6% (0.1)	19.4% (4.4*)	7.8% (−2.2*)	1.9% (−5.3*)	1.3% (1.3)

*Note*: Asterisks show significant differences between zones (values > ±1.97). Negative results indicate that a social behavior was performed less than expected by chance, while positive results indicate that a social behavior was performed more than expected by chance.

## 4 DISCUSSION

We found mixed support for our predictions that the platyrrhine species at LSBRS would engage in less social behavior when in forest edge compared to interior regions. In accordance with predictions, the Central American spider monkey showed a lower rate of social behavior when in forest edge, but the rate of social behavior for the other platyrrhine species did not differ. As predicted, the white‐faced capuchin monkey performed different types of social behavior than expected based on chance when in forest edge, but not all of those predicted, and other monkey species showed no differences.

We expected all three monkey species at LSBRS to demonstrate lower rates of social behavior when in forest edge compared to interior, corresponding with findings from other human‐altered sites (Clarke et al., 2002; de Vries, 2017; Irwin, 2007; Juan et al., 2000; McKinney, 2010, 2019; Ukizintambara, 2010). In line with these results, we found that Central American spider monkeys did have lower rates of overall social behavior when in forest edge zones at LSBRS, although the types of social behaviors (allogrooming, playing, agonism, vocalizing, copulation) performed across zones did not differ. Our results show differences between forest zones, similarly to findings on spider monkey social behavior from El Zota Biological Field Station, Costa Rica, but contradict findings from Lacandona rainforest, Mexico (Chaves et al., 2011; Lindshield, 2006). Spider monkeys at Lacandona rainforest did not differ in the proportion of time engaged in social behavior across fragmented and continuous forest zones (social behavior classified as allogrooming, playing, agonism, Chaves et al., 2011), while monkeys at El Zota showed more social behavior in disturbed compared to continuous forest regions (social behavior not defined, Lindshield, 2006).

At LSBRS, we used a broader definition of social behavior (this study: allogrooming, playing, agonism, vocalizing, copulation), which may have impacted results. Spider monkeys may have performed fewer social behaviors overall when in forest edge due to energy‐saving behavior in this less rich habitat area at LSBRS (Bolt et al., 2018, 2019). Similarly to other frugivores (e.g., greater dwarf lemur [*Cheirogaleus major*], Lehman, Rajaonson, et al., 2006; rufous brown lemur [*Eulemur fulvus*], Lehman, 2007), spider monkeys avoid the edge and prefer forest interior in other habitats (e.g., red‐faced spider monkey [*Ateles paniscus*] in Guyana and Brazil, Lehman, 2004; Lenz et al., 2014), likely due to their reliance on fruit from mature feeding trees, which are found in greater density in forest interior habitats (Kapos et al., 1993; Laurance, 1991, 2000; Lenz et al., 2014). Spider monkeys also prefer interior habitats due to their preference for travel in high canopy, meaning that they are not able to locomote as efficiently in forest edge due to fewer large trees and decreased canopy connectivity (Kapos et al., 1993; Lenz et al., 2014). For these reasons, anthropogenic edge can be considered a less rich habitat for spider monkeys (Lenz et al., 2014). At LSBRS, although spider monkey habitat use did not differ across edge and interior zones (Bolt et al., 2018, 2020b), monkeys may modify travel and feeding activity budgets to compensate for the reduced amount of food available in the forest edge and spend more time feeding, as has been found at for spider monkeys in fragmented regions of Lacandona rainforest (Chaves et al., 2011). While potential differences in overall activity budgets have not yet been assessed for the spider monkeys at LSBRS, social behavior rate differs, with spider monkeys reducing all types of social behavior when in edge regions, potentially to increase foraging time. Spider monkeys at LSBRS did not allocate social behavior type differently when in different habitat zones at LSBRS, suggesting that they may rely solely on overall activity budget adjustments to compensate when in edge areas of reduced habitat quality.

Conversely, differences in spider monkey social behavior rate at LSBRS may not be biologically meaningful. With spider monkey social behavior occurring infrequently (4.2% of scans) during our 236 h of sampling, our initial results here show statistical significance but not large differences in social behavior rates. A larger sample size of behavioral data is needed to clarify trends. Further research on spider monkey social behavior at LSBRS and other human‐altered sites will elucidate the biological relevance of our findings.

In contrast to our findings for spider monkeys, overall social behavior rate for white‐faced capuchin and mantled howler monkeys did not differ across edge and forest interior zones at LSBRS. Our results for capuchin monkeys may be due to species‐specific edge preferences. Capuchins have neutral or positive responses to forest edge (brown capuchins [*Sapajus apella*], Lenz et al., 2014; white‐faced capuchins, Bolt et al., 2018, 2020b; Tinsley Johnson et al., 2020), likely due to their flexible diets and an abundance of key feeding tree species (Tinsley Johnson et al., 2020) as well as preferred foods such as insects (Lenz et al., 2014), and fruit from invasive, photophilic plants (Bolt et al., 2020b) in forest edges. While capuchins can thrive in forest edges and other degraded forest areas (Tinsley Johnson et al., 2020), they require consistent access to a water source to do so (Fedigan & Jack, 2001). At Santa Rosa National Park, Costa Rica, white‐faced capuchins demonstrated higher levels of agonism during the dry season when near watering holes (Rose & Fedigan, 1995), suggesting that resource availability may change capuchin social behavior rates across landscape zones. At LSBRS, the La Suerte river runs throughout the forest including through edge zones, meaning that capuchins in most parts of the forest edge have consistent access to water across seasons. Their social behavior may not show differences in overall rate across zones due to the abundant water resources at LSBRS mitigating rate differences that would otherwise be present (McKinney, 2010). Forest edges at LSBRS are also likely influenced by “edge additivity” (Malcolm et al., 2017), where fragments are simultaneously influenced by more than one type of edge, and/or fragments are small enough that multiple edges influence locations within. The presence of the La Suerte river as well as the shape of LSBRS may make it susceptible to edge additivity (Malcolm et al., 2017), which would further obscure trends in capuchin social behavior across forest zones. Further research on capuchin social behavior at LSBRS, including differentiating anthropogenic edge zones from natural riparian edge zones and combined anthropogenic and riparian edge zones (Bolt et al., 2020b; Schreier et al., 2022b), will help elucidate any differences that have been obscured by edge additivity in the present study.

Despite the lack of overall differences in social behavior rate, capuchins varied the types of social behaviors in different forest zones at LSBRS. Capuchins performed more play and fewer agoniztic and vocalization behaviors than expected when in forest edge, and the converse when in forest interior. Following results from white‐faced capuchins across human‐disturbed versus less disturbed landscape zones at Curu Wildlife Refuge, Costa Rica (McKinney, 2010), we expected capuchin monkeys at LSBRS to similarly perform more affiliative (allogrooming, play) and fewer aggressive (agonism) behaviors in forest edge compared to interior, but we found mixed support for this idea. While capuchins at LSBRS did perform more play and less agonism in forest edge in accordance with predictions, they did not vary their amount of allogrooming across zones. Capuchins also vocalized less than expected when in forest edge but did not differ in the amount of copulation across zones. McKinney (2010) suggested that capuchins showed changes in social behavior across different areas of the same forest at Curu Wildlife Reserve due to habitat degradation causing higher long‐term stress levels in monkeys, and this impacting social behavior. Conversely, differences in capuchin social behavior at LSBRS may not be caused solely by habitat differences across forest zones, but may also be impacted by other non‐mutually exclusive factors including differences in group size, number of adult males, and level of intergroup agonism, which impact capuchin social behavior at other sites (McKinney, 2010; Perry, 1997; Rose, 1994; Tinsley Johnson et al., 2020) but were not assessed in this study.

Capuchins may also be performing fewer agoniztic and vocalization behaviors when in edge zones due to increased predation pressure, as has been suggested to explain differences in howler monkey long calling behavior at LSBRS (Bolt et al., 2019, 2021a; Schreier et al., 2022a). Due to their small body size, capuchins have higher predation risk than other monkey species at LSBRS and would be at especially high risk in the exposed forest edge (Cheney & Wrangham, 1987; Di Fiore, 2002). A robust guild of predators, including ocelots (*Leopardus pardalis*), margays (*Leopardus wiedii*), and spectacled owls (*Pulsatrix perspicillata*), is present in both forest edge and interior zones at LSBRS, indicating a high level of overall threat at this site (Bolt et al., 2023a). Capuchins may vocalize less and perform fewer noticeable physical altercations (i.e. agonism) with one another in forest edge to decrease chances of attracting predator attention. However, the greater amount of play behavior and expected amount of allogrooming for capuchins while in forest edge suggests that affiliative social behaviors are also abundant in this preferred forest zone. Overall, our findings support the idea that capuchins are behaviorally flexible when faced with differences in habitat quality (McKinney, 2010; Tinsley Johnson et al., 2020), although further research at LSBRS is needed to address the myriad factors that may be impacting social behavior across landscape zones in this complex species.

Our results for mantled howler monkeys indicate that they are able to survive in the degraded habitat at LSBRS with minimal modifications to social behavior, although previous research on mantled howler monkey social behavior in disturbed habitat zones at other sites demonstrated some degree of flexibility in social behavior. At Los Tuxtlas, Mexico, and Hacienda La Pacifica, Costa Rica, monkeys performed fewer social behaviors in deforested areas (Clarke et al., 2002; Juan et al., 2000), while at Curu Wildlife, Refuge, Costa Rica, monkeys performed social behavior at lower rates (McKinney, 2019). When contextualized in these previous findings, our results suggest that howler monkeys at LSBRS do not modify rate or type of social behavior across habitat zones because they do not need to. Alternately, howler monkeys at LSBRS may not adjust social behaviors because they are not able to, attesting to the inflexibility of this species’ social adaptations in disturbed environments. This behavioral inflexibility in degraded forest is supported by findings from mantled howler monkeys in Los Tuxtlas, Mexico (Estrada et al., 1999b) as well as black howler monkeys at Monkey River, Belize (Horwich et al., 1993; Pavelka & Knopff, 2004). Both black and mantled howler monkeys at these sites did not alter social behavior or activity budget even when consuming higher‐quality diets, suggesting that they may be limited in their ability to alter behavior (Estrada et al., 1999b; Horwich et al., 1993; Pavelka & Knopff, 2004). Regardless of the cause of the behavioral rigidity observed in the howler monkeys at LSBRS as well as at other sites, mantled howler monkeys at LSBRS are able to survive at high density in degraded edge areas while making minimal adjustments to social behavior, demonstrating the survival ability of this species at this site for now. Schreier et al. (2022b) found that howlers at LSBRS modified their overall activity budgets in anthropogenic zones, but these differences were only apparent when natural riparian edge zones were included in analysis, in addition to anthropogenic edge zones. Further research on howler monkey social behavior at LSBRS, including examination of how proximity to the La Suerte river affects social behavior, will allow us to more fully understand howler monkeys' social adaptations.

## 5 CONCLUSIONS

Overall, we found that spider and capuchin monkeys at LSBRS modified their social behavior when in forest edge compared to interior habitat, while howler monkeys’ social behavior remained consistent across forest zones. Central American spider monkeys had a lower social behavior rate and white‐faced capuchin monkeys had different social behavior types when in forest edge zones. Investigating how monkeys change their behavior in response to anthropogenic deforestation is important for gaining a more nuanced understanding of how each species adjusts and survives when in various types of degraded habitat areas. Any human‐caused alteration of primate behavior is a conservation concern and should be avoided in the interest of general animal well‐being (Bolt et al., 2020a). With social behavior being a key adaptation for group‐living mammals (Kappeler et al., 2013), its incorporation into edge effects studies is important. Our results for two of three platyrrhine species at LSBRS demonstrate altered social behavior in anthropogenically‐impacted forest zones, providing further evidence of the detrimental effects of human‐caused forest degradation on primates.

## AUTHOR CONTRIBUTIONS

**Laura M. Bolt**: Conceptualization (equal); data curation (equal); formal analysis (equal); investigation (equal); methodology (equal); project administration (equal); supervision (equal); visualization (equal); writing—original draft (lead). **Jenna L. Owens**: Data curation (equal); investigation (equal); writing—review and editing (supporting). **Madison Taylor Grant**: Data curation (equal); investigation (equal); Writing—review and editing (supporting). **Elizabeth M. C. Coggeshall**: Data curation (equal); investigation (equal); writing—review and editing (supporting). **Dorian G. Russell**: Data curation (equal); investigation (equal); writing—review and editing (supporting). **Carrie Merrigan‐Johnson**: Data curation (equal); investigation (equal); writing—review and editing (supporting). **Zachary S. Jacobson**: Data curation (equal); investigation (equal); writing—review and editing (supporting). **Zachary T. Schmidt**: Data curation (equal); investigation (equal); writing—review and editing (supporting). **Francesca V. E. Kaser**: Data curation (equal); formal analysis (equal); software (equal); visualization (equal); writing—review and editing (supporting). **Amy L. Schreier**: Conceptualization (equal); data curation (equal); formal analysis (equal); investigation (equal); methodology (equal); project administration (equal); supervision (equal); visualization (equal); writing—original draft (supporting).

## CONFLICT OF INTEREST STATEMENT

The authors declare no conflicts of interest.

## ETHICS STATEMENT

Our research was conducted with the permission of the Molina family and met the legal requirements of Costa Rica. Our research adheres to the American Society of Primatologists (ASP) Principles for the Ethical Treatment of nonhuman primates and was approved by Regis University's Institutional Animal Care Committee (IACUC permit #17‐006).

## Data Availability

The data that support the findings of this study are available in figshare at 10.6084/m9. figshare.24994685
